# A Novel Mobile App (“CareFit”) to Support Informal Caregivers to Undertake Regular Physical Activity From Home During and Beyond COVID-19 Restrictions: Co-design and Prototype Development Study

**DOI:** 10.2196/27358

**Published:** 2021-10-01

**Authors:** Kieren J Egan, William Hodgson, Mark D Dunlop, Gennaro Imperatore, Alison Kirk, Roma Maguire

**Affiliations:** 1 Department of Computer and Information Science University of Strathclyde Glasgow United Kingdom; 2 School of Psychological Sciences and Health University of Strathclyde Glasgow United Kingdom

**Keywords:** physical activity, Android, COVID-19, intervention, co-design, exercise, app, development, support, caregiver

## Abstract

**Background:**

Informal caregivers, or carers (unpaid family members and friends), are instrumental to millions worldwide for the ongoing delivery of health and well-being needs. The risk of crisis points (eg, hospitalizations) for caregivers increases with the absence of physical activity. The COVID-19 pandemic is highly likely to have increased the risk of crisis points for caregivers by increasing the amount of time spent indoors due to shielding and lockdown restrictions. Thus, accessible evidence-based tools to facilitate physical activity for caregivers indoors are urgently needed.

**Objective:**

The aim of this study was to co-design and develop a novel mobile app to educate and support carers in the undertaking of regular physical activity at home during and beyond COVID-19 restrictions via integration of the transtheoretical model of behavior change and UK physical activity guidelines.

**Methods:**

We co-designed a mobile app, “CareFit,” by directly involving caregivers, health care professionals, and social care professionals in the requirements, capturing, and evaluation phases of three Agile Scrum design and development sprints. Seven participants representing multistakeholder views took part in three co-design sessions, each of which was followed by a development sprint. Requirements for CareFit were grounded in a combination of behavioral change science and UK government guidelines for physical activity.

**Results:**

Participants identified different barriers and enablers to physical activity, such as a lack of time, recognition of existing activities, and concerns regarding safely undertaking physical activity. Requirements analysis highlighted the importance of simplicity in design and a need to anchor development around the everyday needs of caregivers (eg, easy-to-use video instructions). Our final prototype app integrated guidance for undertaking physical activity at home through educational, physical activity, and communication components.

**Conclusions:**

Integrating government guidelines with models of behavioral change into a mobile app to support the physical activity of carers is novel. We found that integrating core physical activity guidelines into a co-designed smartphone app with functionality such as a weekly planner and educational material for users is feasible. This work holds promise to fill the gap of effective physical activity solutions for caregivers both during and beyond the COVID-19 pandemic. Further work is now needed to explore the feasibility, acceptability, and usability of the approach in real-world settings.

## Introduction

Informal caregivers or carers—those providing unpaid care for friends or family—constitute a vital lifeline to millions of people worldwide. In the United Kingdom alone, there are an estimated 6.5 million carers, and across Europe, up to 80% of all long-term care is understood to be delivered by carers [1,2]. Although some carers benefit and achieve a sense of fulfillment from caring roles [3], there is now strong evidence that caregiving may have an adverse impact on health and wellness both in the short and long term [4,5]. Preventable crisis points (eg, hospitalizations, significant worsening of mental or physical health, irreversible changes to caring circumstances) are commonplace (even in the absence of COVID-19) and frequently cause irreversible deterioration in health for the carer and those cared for [6,7]. As the global population ages, and the health and social care workforce shrinks [8], it appears inevitable that the reliance placed on caregivers will only increase. A public health priority is to raise quality of life and prevent crisis points. Furthermore, the COVID-19 pandemic substantially increased pressures and time spent at home, and reduced opportunities and motivations for physical activity [6,9,10].

The unmet needs of caregivers are considerable and diverse. There have been many innovations in recent years to aid caregivers in areas such as support, care coordination, telehealth/diagnostics, and digital care delivery [11]. Solutions aimed at caregiver support have mainly focused on targeting mental health (eg, burden, anxiety, depression) through face-to-face, telephone, and digital interventions [12,13]. Less established solutions are those aimed at improving physical health. Systematic review work in this area identified only 14 studies to date [14], with interventions mainly delivered face to face and/or by telephone-based approaches. Across these studies, improvements were observed in physical activity levels, distress, well-being, quality of life, and sleep quality. Such targeted solutions are yet to make the “leap” into the digital spectrum and mass impact potential of smartphone apps.

**Figure 1:**
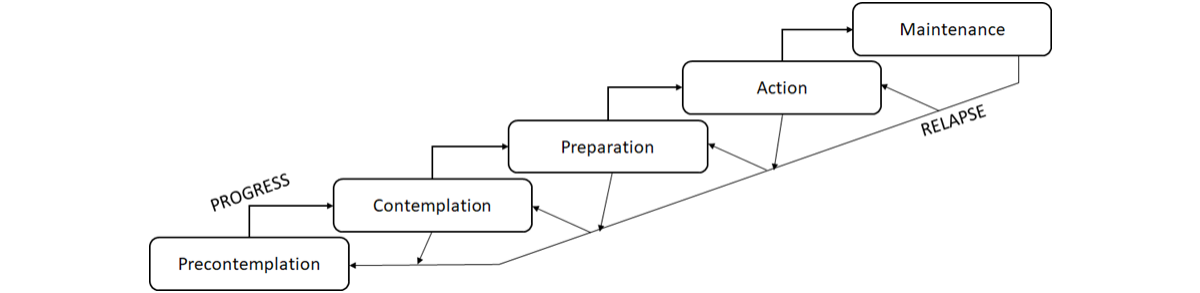
Overview of the transtheoretical model across the different stages of precontemplation, contemplation, preparation, action, and maintenance alongside relapse.

The potential of the digital spectrum is now emerging for all populations (eg, automated data collection, machine learning, augmented reality), and there are key questions as to whether vulnerable groups such as informal caregivers will also be able to enjoy the benefits. Advantages could include simply raising awareness of physical activity guidelines through mobile apps (such as those from the United Kingdom, which suggest a variety of different types of activities per week according to age group). More sustainable and greater impacts may also be realized through using evidence-based models of behavioral change. The well-established transtheoretical model (TTM) of behavioral change [15,16] postulates that the more sustainable changes in behaviors are those that are altered habitually and through a cyclical process of specific stages (see Figure 1). However, it remains to be explored precisely how to design and integrate a solution capable of translating such key messages in a feasible, acceptable, and usable manner for more vulnerable groups such as caregivers.

Recent survey data suggest that (even in the absence of COVID-19) 81% of carers are not able to perform as much physical activity as they would like [17]. There is therefore an imperative need to continue researching innovations for caregivers, and to explore what empowering evidence-based tools could be delivered at home both during the COVID-19 pandemic and beyond. We here present a rapid response project to produce a novel evidence-based mobile app designed to empower caregivers to undertake regular physical activity at home during (and beyond) the COVID-19 outbreak. We designed our app, “CareFit,” with a co-design team of user experts, and using robust and well-established scientific knowledge (eg, the TTM [15,16], government guidelines [18], and sports and exercise specialist knowledge).

## Methods

### Recruitment, Consent, and Ethical Approval

Participants were recruited using convenience sampling (connections across both Carers Scotland and the University of Strathclyde). We aimed to identify 6-8 participants to a co-design group to maximize the depth of conversation achievable with our discussions [19]. We contacted three known professionals who had an interest in caregivers. Caregivers were recruited specifically though links with Carers UK (Scotland), whereby (a few/targeted) local carer centers across Scotland were asked to approach carers who would be suitable for the study and interested in being part of a working group. All invited participants accepted the offer to take part in this work. Inclusion criteria were that participants were aged 18 years and over and interested in contributing to current knowledge of digital innovations for caregivers. Individuals were asked to commit no more than 7 hours in total to the co-design process. The co-design sessions took place between July and August 2020 (ie, during the COVID-19 pandemic). The University of Strathclyde Ethics Committee approved the study protocol. As per standard ethical procedures, each member of the group signed an individual consent form after being given an information sheet and opportunity to ask any questions about their overall involvement in the study.

### Study Design

**Figure 2:**
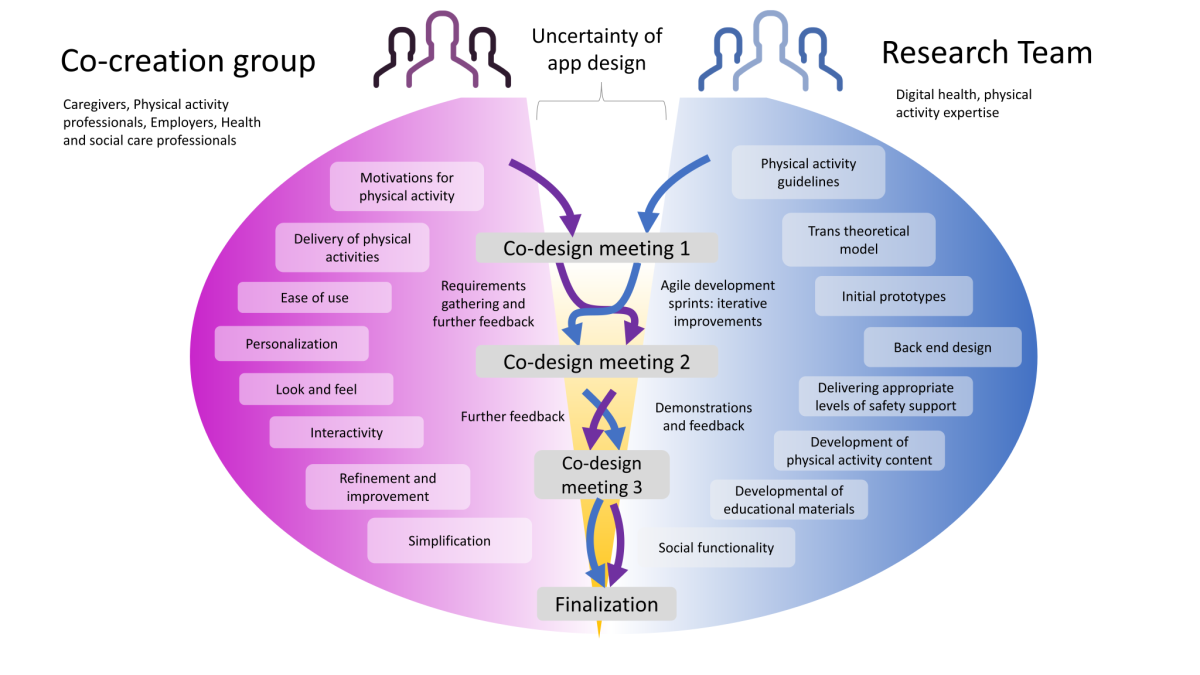
Overview of the co-design process across the three co-design meetings.

We developed CareFit using an Agile Scrum co-design methodology [20,21] (see the App Development and Testing section below), and had to work within national and local restrictions imposed by COVID-19 pandemic measures, which represented a considerable challenge given the face-to-face nature of traditional co-design sessions. We viewed our co-design participants as architects and partners of this work (see Figure 2), as outlined by Sanders and Stappers [22]: “creativity of designers and people not trained in design working together in the design development process.” Our stakeholders consisted of carers, employers, physical health experts, and health care professionals. This multidisciplinary team was involved throughout the design and evaluation stages of the co-design process [20,22]. In total, three versions of the app were developed iteratively, and the final version of the app was released and evaluated by our participants (caregivers and caregiver-related professionals). Our focus was on people in the contemplation or preparation stages of the TTM, which includes those only thinking about being more active, and those who have thought about and taken some steps to becoming more physically active. Our goal was to help participants form and regularly “action” intentions to be more active. In terms of the software/tools used, we conducted three co-design video call sessions (using Zoom and simulations of notice boards/post-its [MURAL]), and complemented collective discussions with three individual questionnaires (incorporating around 20 to 30 questions on Qualtrics online software) as a basis for design sprints. If a participant could not attend group meetings, one-to-one calls were offered as an alternative. See Table 1 for an overview of the meetings. The time immediately after our co-design meetings was dedicated to our development “sprints,” each lasting 2-3 weeks.

**Table 1:** Overview of the co-design meetings.

Sprint (number of questionnaires completed)	Focus/aim	Detail of meeting used to guide sprint
1 (N=5)	To critique and present a simple initial app prototype; to collectively present the principles of the transtheoretical model (TTM) and the UK government national physical activity guidelines.	We explored the topics of motivation, goals, physical activity guidelines, delivery options, health, and safety. We also explored “keep, lose, change,” and asked our participants to prioritize needs according to the MoSCoW^a^ methodology.
2 (N=6)	To review the feedback from meeting 1 and progress during design sprint 1.	We explored how to deliver details within the educational, physical activity, and communication components, including the “keep, lose, change” format. We presented future options of the physical activities using videos and subsequent feedback.
3 (N=6)	To review and finalize the app design in preparation for a 3-week real-world study.	Final, detailed discussion on the presentation of the revised app developed, and further discussion of the education, physical activity, and communication sections.

^a^MoSCoW: “Must Have, Should Have, Could Have, Won't Have this time” prioritization.

### App Development and Testing

The CareFit app was developed for Android (versions 7 to 10). The app was mainly developed in Java, with the exception of the education section, which was developed in HTML/CSS/JavaScript and integrated into the main app. Extensive unit and user testing was undertaken using Android phone simulators and a range of different Android physical devices of different ages, specifications, and display sizes. Test versions of CareFit were distributed to users through online emulators in advance of the co-design meeting sessions to improve the requirements-capturing discussions and involve target users in the development process. CareFit was developed using the Agile Scrum methodology. Agile Scrum is an iterative software development process in which software development takes place in short and fast periods of development formally defined as sprints. Before each development sprint, requirements from the previous sprint are improved or new requirements were captured using feedback given to developers by users. We categorized functional and nonfunctional requirements using the FURPS+ (Functional, Usability, Reliability, Performance, where the “+” is used to indicate additional requirements such as programming language and other constraints) approach [23]. Such requirements were guided throughout by our co-design team using MoSCoW (“Must Have, Should Have, Could Have, Won't Have this time”) [21] and “keep, lose, change” [22] methodologies, and informed by the TTM [15]. Our design supported users by enabling them to report errors and crashes easily through a dedicated email address. The email address was displayed on the main screen of the app at all times and, where possible, we provided an immediate response to users with technical issues. CareFit had the following “+” requirements: being developed for Android OS (support ranged from Android 7.0 known as “Nougat” to Android 10 known as “Pie”).

### Data Handling and Prioritization

The structure of co-design sessions consisted of an online white board (MURAL), online conference calls (Zoom), and online questionnaires (Qualtrics). All of our online meetings involved the presentation of slides and/or prototype mockups/video “walkthroughs.” The first development sprint involved requirements-capturing using the MoSCoW methodology prioritization method, which ranks requirements as “must have,” “should have,” “could have,” or “won’t have” [24]. During the co-design and sprints 1 and 2, we also used the “keep, lose, change” approach to offer our participants freedom to decide on fundamental aspects of the app where required [25]. Whenever the majority of the group expressed clear and strong preferences, these were integrated in the app design. There was a small number of occasions where user suggestions conflicted with physical activity guidelines. Any discrepancies to MoSCoW preferences are explained within the text. When the co-design group did not reach consensus, the academic team reviewed and reached a final decision. For qualitative data, quotations were examined by two researchers (BH, KE) who analyzed data and identified core themes. Disagreements were discussed with a third researcher (RM).

## Results

### Co-design Group

Our co-design group consisted of four different stakeholder groups: caregivers (n=4), a health care professional (n=1), an expert in physical activity (n=1), and an employer representative who supported caregivers on a regular basis (n=1). Our sample included six women and one man. All our participants resided in Scotland. Our three group meetings involved discussions about all aspects of the app design and were hosted by three researchers including our lead developer. Follow-up to group sessions involved the cumulative delivery of more than 100 questions delivered in the format of online questionnaires. This work was undertaken as a rapid response to the COVID-19 pandemic, and was carried out over 6 months (July to November 2020).

### Co-design Meeting 1 and Design Sprint 1

As part of co-design meeting 1, we presented a simple exercise app based on National Health Service guidance on exercises without integration of any behavioral change models or national guidelines (see Multimedia Appendix 1 for an example questionnaire). Participants highlighted that barriers and enablers to physical activity included lack of time, motivation, safety, recognition of achievements, and a need for personalization (see Textbox 1).

We asked participants what they would like to “keep, lose, (or) change” after reviewing a basic prototype that contained some physical activity exercises of different intensities, a very basic reminder system, and text instruction of exercises with an accompanying timer. Participants wished to “lose” the timer for strength exercises and “keep” aspects regarding icons. “Change” included the addition of videos to demonstrate safe ways to complete exercises, and to find ways to capture progress (eg, “include planner and progress chart” and “add a video to demonstrate a safe way of completing exercises”). We further explored how best to deliver safety information to participants. In total, 60% of users preferred a disclaimer about a risk of injury and/or a summary about safety only on the “first login.” The addition of instructions for safe exercise on “every login” was supported by 60% of users.

**Barriers and enablers discussed during our first co-design session with representative quotes for each theme.**

**Enablers**

- Incorporate daily living activities as physical activity opportunities
- Explore user support
- Motivational strategies
- Provide users with physical activity advice and safe practice

Example quote, “Time: making it short and simple and able to do in their own time; reminders to motivate; peer support.” [Participant 1.5]

**Barriers**

- (Lack of) peer support
- Poor mental health
- Lack of education
- Changing definition of wellness
- Lack of recognition
- Lack of individualized approaches
- Support missing to receive coping strategies

Example quote, “Time constraints, financial pressures, physical impact of caring (eg, back injury), emotional barriers (eg, guilt over leaving loved one), lack of respite opportunities, ineffective coping strategies, lack of motivation (exacerbated by depression).” [Participant 1.3]

Participants were primarily interested in the app supporting delivery of physical activity elements (47.8%) and secondarily interested in the education and social/community components (31.8% and 20.4%, respectively). Participants stated a desire for education and physical activity information to be displayed graphically (ie, less text-based). Use of icons, graphs, and videos was a particularly popular approach (40% of participants stated visual elements were a “must have” feature, with 20% stating that audio and video elements were also “must haves”). In terms of personalization, 20% of the participants stated that use of the first name of the carer was a “must have,” whereas none of the participants classed displaying the name of the person cared for as a “must have” feature.

**Figure 3:**
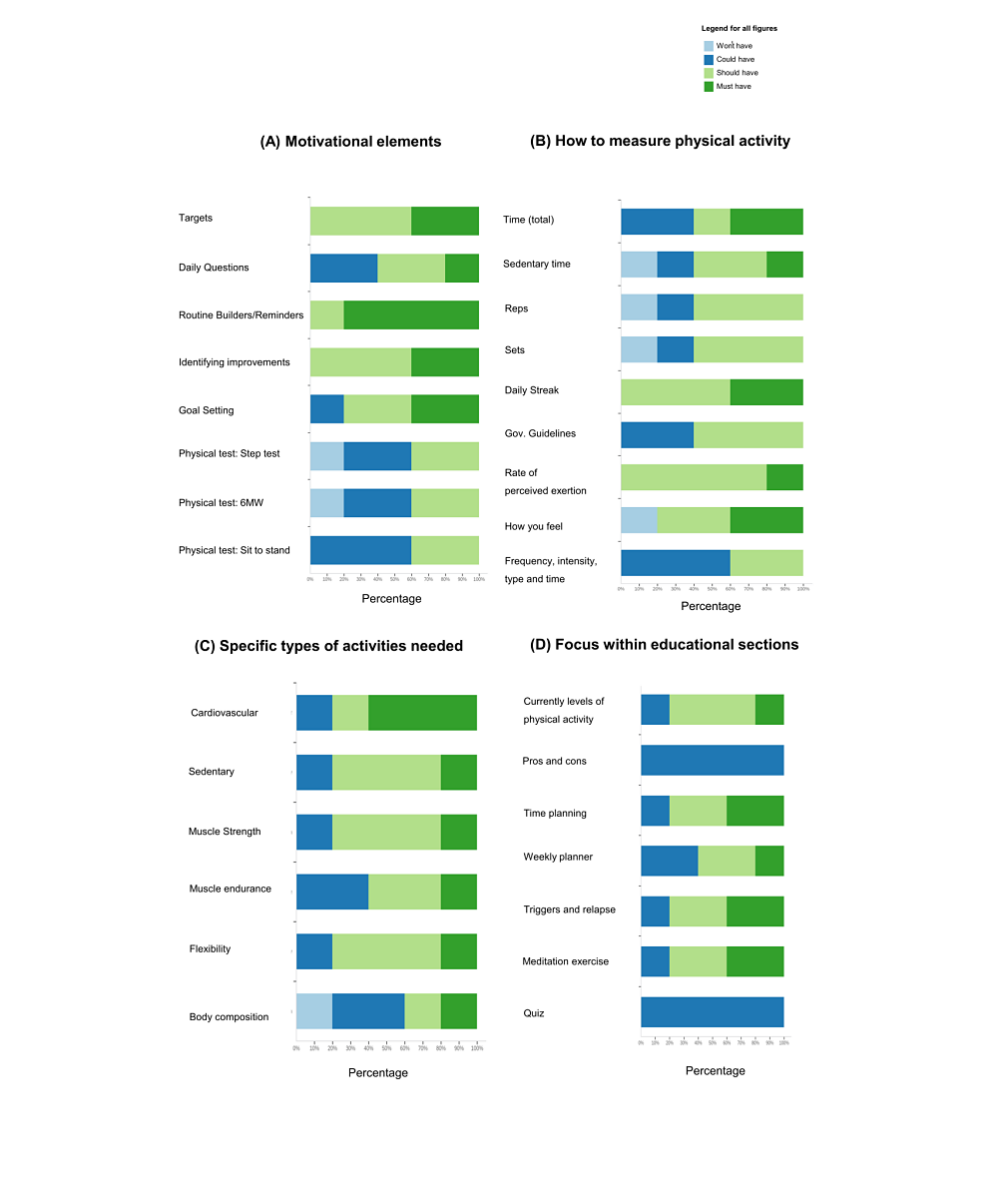
Feedback received using MoSCoW methodology across motivational elements (A, top left), measuring physical activity (B, top right), specific types of activities needed (C, bottom left), and focus within education sections (D, bottom right). Abbreviations used: 6 Minute walk test (6MW), Government Guidelines (Gov. guidelines).

For the physical activity/motivation elements, most of the participants (80%) stated that routine builders were a “must have” component of the app (see Figure 3A). Key requirements for users (ie, 40% of users stating that these were “must have”) included goal/target setting and identifying improvements (Figure 3A). Participants were presented with three potential designs (Figure 3B) for functionality to measure physical activity progress. Despite reacting positively to the idea, there was no consensus on precisely how this could be implemented. Participants recognized the need for caregivers to undertake different types of physical activities, with cardiovascular physical activity identified as the greatest need (60% of participants stating this as a “must have” feature; Figure 3C). Other prominent features included muscle (endurance and strength), flexibility, and breaking up sedentary behaviors (20% of participants respectively stated these were “must have” elements).

**Figure 4:**
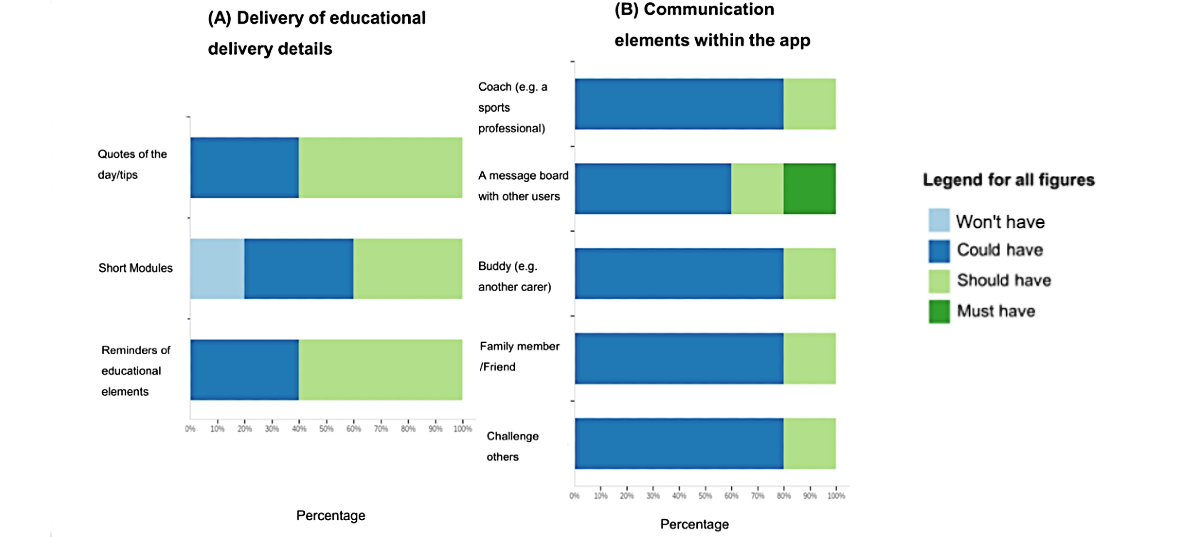
Feedback from participants using the MoSCoW method for (A) delivery method for educational details and (B) communication elements within the app.

Respondents were divided as to what the educational elements should look like. There were no clear interactive features that were recognized as a requirement for all users (Figure 3D); however, there was some preference to add functions such as “meditation exercises,” “triggers and relapse prevention exercises,” and elements regarding “time planning.” Some participants (40%) thought that tips or quotes of the day were requirements that the app “could have,” as opposed to 60% who thought the app “should have” these elements (Figure 4A). There was no consensus on how communication elements of the app could be delivered. A variety of formats were suggested, such as the presence of a coach, a message board, and challenging other users (Figure 4B).

### Implementation of Design Sprint 1

To implement the requirements gathered in co-design meeting 1, we reviewed the data gathered as a research group and improved our app accordingly (see Table 2). The majority of user requirements (eg, routine builder, time planner) were implemented through the development of a weekly planner, physical activity content, and education and communication elements. Physical activity plans were simplified from the UK guidelines as much as possible so that “muscle and balance” could encompass aspects of muscle strength and endurance alongside flexibility. Educational materials were influenced by both carer needs and previous paper-based resources developed for the diabetes field (adapted for use with caregivers). We took an academic decision not to include GPS functionality step counting, as carers may not always carry their phones and therefore could potentially lose recognition for the physical activity undertaken. There are also many existing apps that focus on this type of physical activity (eg, running, walking). We still planned to incorporate outdoor exercise aspects into the app. We also took the decision to support carers using the app with a user guide in addition to the guidance delivered in educational sections. This represents our aim to develop an app without requiring significant external training to use it.

**Table 2:** Requirements identified and developed within co-design stage 1.

Requirements		Development/implementation details
**Physical activity needs**		
	Need to develop a simple evidence-based physical activity plan^a,b^	An easy-to-use planner where an entire week would be visible, ideally reflecting (1) cardiovascular, (2) muscle and balance, and (3) sedentary breakers according to the UK physical activity guidelines. This “weekly planner” was the cornerstone of the app’s physical activity functionality where users could make, revise, view, and review their plan for the week(s) ahead.
	Users would like to record any cardiovascular activity (ie, at home and outside)^a,b^	For cardiovascular activities, we built a simple dialogue system that could record time and intensity. We also incorporated “active living” activities through a drop-down menu for adding further detail.
	Muscle and balance simplicity^a,b^	We devised a system that incorporated 3 to 5 different muscle and balance activities (with the precise content yet to be determined), allowing personalization.
	Underline importance of health and safety^a,b^	Users are supported with information about how to undertake safe exercises both through an initial information and disclaimer screen, alongside some brief information within each physical activity video.
	Capturing sedentary activity^a,b^	Users can optionally record sedentary activity.
Educational needs: Increase awareness of the activity guidelines and behavioral change^a,b,c^		Initial educational content was developed on PowerPoint for subsequent transfer to the app. The format follows the activity consultation (built in part from existing resources within the group for diabetes, and includes interactive elements based on the TTM^d^).
Communication needs: Flexibility on how social media/messaging could be implemented^a^		As “communication” was a lower priority feature, we remained open to comments and considerations from the group. Our plan was to be agile in our development. We concentrated our efforts primarily around exploring links to social media and message boards.
**Look and feel**		
	The app should be simple to navigate and personalized.^a,e^	Controls were clearly marked with labels. For the educational section users could choose the font size to facilitate reading.
	App colors that are familiar/associated with trust to users should be used.^a,e^	User interface was designed to keep the different sections of the app compartmentalized both visually and functionally, while the look and feel of the app was kept consistent; by using different colors and clear labels, users were always kept aware of which section of the app they were in.
	The components around education and physical activities should be clearly distinct.^a^	To improve user experience, the educational section was implemented in HTML/JS/CSS as this section was primarily text-based.

^a^Based on co-design discussions.

^b^Based on UK activity guidelines.

^c^Based on models of behavioral change.

^d^TTM: transtheoretical model.

^e^Based on user design principles.

### Co-design Meeting 2 and Design Sprint 2

#### Overview

We presented a revised prototype to participants based on feedback from design sprint 1. Feedback was generally positive; in particular, strengths of the work mentioned included the simplicity of design and user-friendliness of the app. Elements suggested to “keep” included the overall app look and feel such as “the simplicity of selecting exercises.” There were no “lose” elements suggested. “Change” elements included themes regarding flexibility/personalization such as *“*the ability to move exercises as things come up on certain days.” Other feedback from participants concerned the colors of the app. Many participants reported that they liked the simplicity of the app (eg, “I think it looks good and it’s concise and to the point.”) To improve usability, participants suggested that many different types of elements could be added, including short and focused educational materials. Some users suggested further improvements to the user friendliness of the app and that a user guide/video introduction could be a useful introduction to the app for carers (eg, “I think make it as user friendly as possible; less is more”).

#### Physical Activity Elements

We asked participants about how they would like reminders to function. There was no consensus about when the best time of the day or week to deliver these would be. Further comments came back from several respondents that more personalization holds value to carers, including “the user could choose this to suit their individual needs such as evenings/weekends.” During co-design meeting 2, we presented to the group an existing short “sedentary breaker” video produced from the University of Strathclyde aimed at staff members. Feedback on the video included that the informality of the activities is a strength and that we should consider increasing the clarity of instructions.

I feel that the style of the video is sufficient but maybe a subtitle on the video of what the carer should also be doing.

Co-design participant 2.4

Good to show in a home setting and using equipment from around the home.

Co-design participant 2.1

The videos are great and also good to have a written description at the side. Would suggest a commentary with each exercise to give advice on exercise and, for example, what muscles you should feel stretching to minimize issues.

Co-design participant 2.5

**Figure 5:**
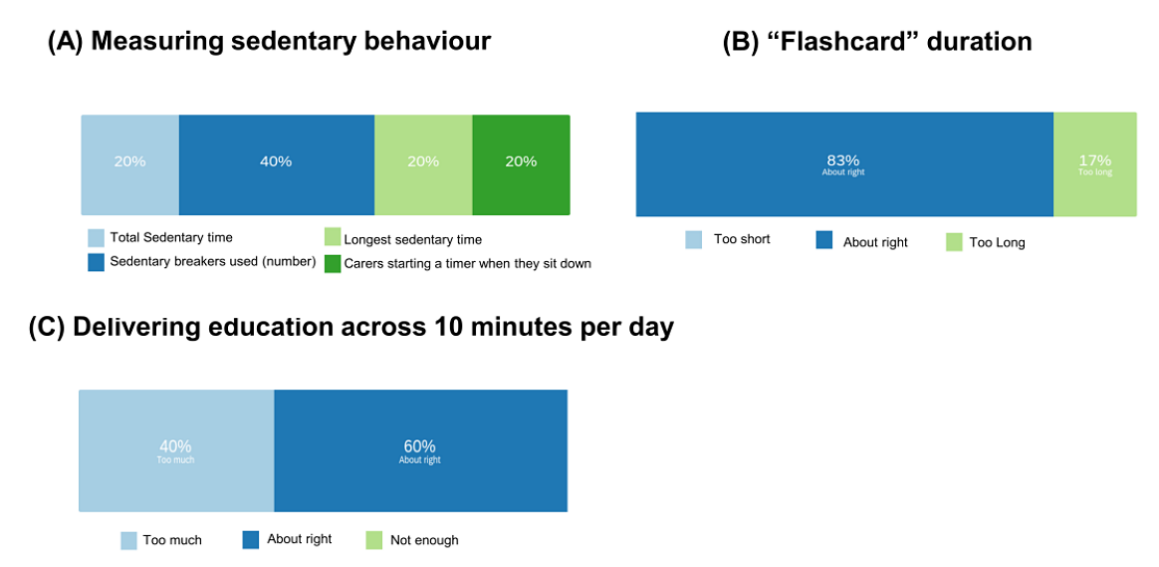
User preferences for components of app design in co-design meeting 2. Participants provided feedback on how best to (A) measure sedentary behavior, (B) deliver flashcard duration, and (C) deliver education across 10 minutes per day.

For selecting a unit of measurement for sedentary achievements, the “number” of sedentary breakers was the top choice from four options (Figure 5A). The majority of our participants (83% of respondents) considered flashcards of around 5 minutes duration to be the most suitable (Figure 5B). Participants requested a wide range of different cardiovascular activities possible (eg, walking the dog, running), some of which could take place outside the home. Participants were also interested to see a broad mixture of different muscle exercises delivered (eg, upper body, lower body), and all of our respondents wanted to see physical activity specific to caregivers incorporated into the overall app design.

#### Educational Elements

We asked our participants if up to 10 minutes a day of interacting with educational materials was feasible for caregivers to carry out; 60% of our participants stated that this was “about right,” compared to 40% of participants who said it was “too much” (Figure 5C). All components of the education fit well with participants’ expectations across the seven original elements proposed: (1) “Introduction,” (2) “Relationships and Physical Activity,” (3) “Managing Time,” (4) “Goals and Rewards,” (5) “Physical Activity and Consequences,” (6) “The Mind and Body,” and (7) “Knowledge Quiz.”

#### Communication Elements

Participants provided feedback that components of the app could be useful to share with friends and family, including goals, activities, barriers, support, and others such as sharing achievements. However, there was no clear consensus on which single aspect would be most useful to share. Participants were interested to integrate their activities with many different platforms, including Facebook (100% of respondents), WhatsApp (83% of respondents), and Twitter/Instagram (50% respondents each).

### Implementation of Design Sprint 2

To implement requirements gathered in co-design meeting 2 (Table 3), the physical activity functionality was refined much further, allowing users to now add up to three activities on any given day of the week in the planner. Cardiovascular activities would be delivered in the app or as “ad hoc,” where details could be recorded in a drop-down menu (eg, walking the dog). Users could also select an intensity of activity ranging from low to moderate to high. We further refined educational sections to incorporate a target of 10 minutes per section, and explored the use of visuals and breaking up text. To support the widest social media integration, we created image-based certificates for user achievements and integrated them through Android’s “Share with” functionality. This meant that our user could share their progress on any social media platform as well as through email or MMS.

**Table 3:** Details of design sprint 2 following requirements identification within co-design session 2.

Requirements			Development/implementation details
**Physical activity needs/themes**			
	Improve the clarity of the videos, including the use of text on the screen^a^	Develop bespoke videos for each of the physical activities supported by the app. This would include text on the screen and audio guides of how to undertake each activity. Videos will cover a wide range of different activities across cardiovascular, sedentary breakers, and muscle and balance work. Three bespoke videos for each activity group will be developed, guided by a physical activity specialist.	
	Participants would like to measure progress in sedentary behavior using number of days ^a^	Implement simple drop-down menu options to record the number of sedentary breakers used per day. This would allow users to set a target for sedentary breakers each day and record progress accordingly.	
	Participants would like to be able to move activities onto the next day^a,b^	A feature will be added to the weekly planner so that users can move an activity forward if not completed at the intended time.	
	Participants would like to set their own reminders as required^a,b,c^	Support users to add reminders for activities as required within the planner. There will also be additional support within the app to allow users to review all reminders set at the same time.	
	Muscle and balance activities need to exercise many different muscle groups within the same activity^a,b^	We would explore the feasibility of developing “flashcards” that would present a sequence of random activities. This could include building more holistic exercise sets within an individual 5-minute video.	
Educational needs: “Lessons” need to last up to 10 minutes per day and deliver the 7 lessons as intended, but the terminology could be off-putting^a,c^			Materials developed for up to 10 minutes a day, and all proposed elements on the app. All educational elements are to be optional and termed “stages” to avoid overly formal language. Development of rules of the education sections, including how to provide consistency of content and delivery.
Communication needs: Allow participants flexibility on the modality of sharing information^a^			Our app must support many different modalities of sharing user progress, and may be more functionally suited to Android system sharing.
**Other**			
	User guide required for participants^a^	User guide will be accessible through the app.	
	Look and feel of the app, including color scheme, need to be revised^a,b,d^	Implement consistent use of logos and color scheme across the different app components based on the activity guidelines and UK National Health Service colors.	

^a^Based on co-design discussions.

^b^Based on UK activity guidelines.

^c^Based on models of behavioral change.

^d^Based on user design principles.

### Co-design Meeting 3 and Design Sprint 3

#### Overview

During this last co-design meeting and resulting sprint, we finalized the app design. We used information already presented to the group and built the final design on key examples (Table 4). Overall, participants responded positively to the design of CareFit’s home screen, most participants (67%) describing it as “very user friendly” and the remaining (33%) describing it as a “little user friendly.” Free-text feedback from participants suggested positive reception of the activity planner. For example, comments described the planner as “easy to understand” or having a “simple layout which is simple to follow”; however, there were some concerns raised by some describing the planner as “busy and hard to follow.” Feedback also highlighted the importance of personalization; for example, participants suggested allowing users to select/design elements of the user interface: “everyone is different and should choose their own color scheme if they can” (co-design participant 3.6).

**Figure 6:**
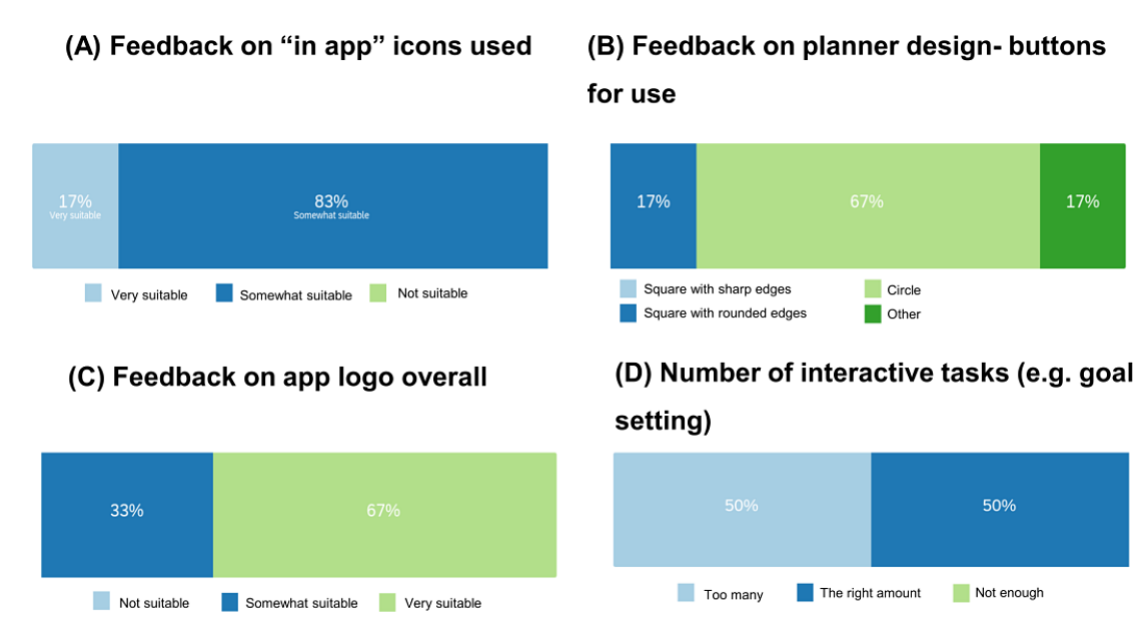
Feedback from participants using the MoSCoW method. (A) Preference for the icon type within the weekly planner; (B) feedback on planner design buttons for use; (C) suitability of our proposed app logo; (D) response on the number of interactive tasks within the educational sections.

**Figure 7:**
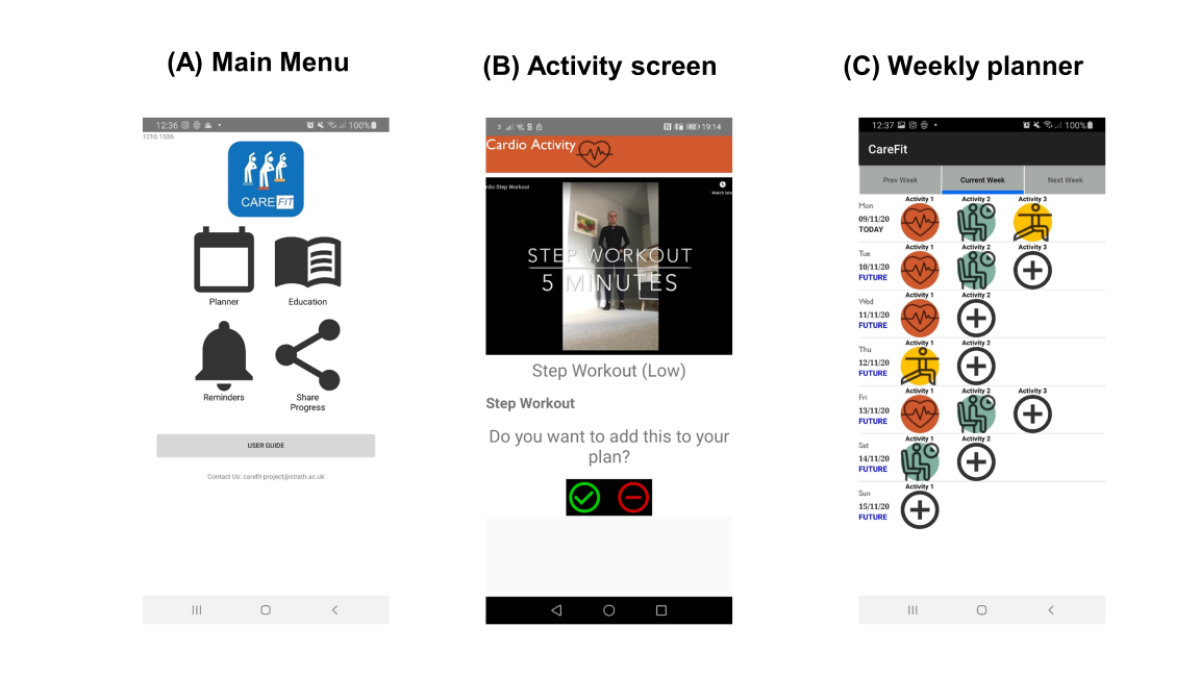
Screenshots from the final app design, including (A) main menu page, (B) example of an exercise page, (C) weekly planner.

After showing participants our proposed design for the user interface, all participants found the icons suitable, including 17% who found it “very suitable” (Figure 6A). Designs presented in discussion included the icons proposed for specific activities. In response, 33% of the participants indicated that use of an “arm flexing” icon was not appropriate for caregivers to signify strength and balance activities. Other feedback indicated that the icons were *“…*simple and easy to recognize and follow.” For the planner (Figure 6B), there was a general preference for rounded circular icons (as opposed to squares or rounded squares). Most participants (67%) thought that the overall app logo design was very suitable (Figure 6C and Figure 7A).

**Table 4:** Details of design sprint 3 following requirements identification within co-design session 3.

Requirements		Development/implementation details
**Physical activity needs/requirements**		
	Participants requested that we alter the icons used (bicep) for muscle and balance	An alternative graphic was selected, more suitable for the carer demographic.
	Participants would like to access physical activities (eg, sedentary behaviors) from within the education sections^a^	Implement a link between the educational and physical activity components to link the two.
	Videos delivered with clarity, supported by text. There was no consensus on university branding; the academic group decided to proceed with videos using the university logo^a^	Videos are supported with slow, clear narration; safety messages; and on-screen text. A link to each video must be accessible within the app delivered when both planning and undertaking activities.
	Participants with physical activity expertise recommended that delivering “muscle and balance” activities with significant variation of targeted areas within each video.	Deliver, record, and integrate videos that support all physical activity types: sedentary activity, cardiovascular activity, and muscle and balance. We will develop 3 short videos (2 to 5 minutes).
	Appropriate measurement of physical activities and progress^a,b,c^	For cardiovascular activities, users measure time and intensity; for sedentary breakers, users measure the number per day; and for muscle and balance, users can measure the number of events. Timing of cardiovascular activities will be measured using a start/stop timer dialogue.
**Educational needs/requirements**		
	As per sprint 2, ensure that educational sections last around 10 minutes or less^a,c^	Split initial educational sections so that there are 8 sections overall: “Introduction” now becomes “Welcome and Introduction” and “Physical activity: Beginners Guide.”
	Increase accessibility of the educational materials ^a,c^	Use more visuals and break up education text
Communication needs/requirements: As per sprint 2		Deliver the ability to share progress across different social media/communication tools.
**Other/look and feel of the app**		
	Participants liked the overall color scheme and logo formats suggested ^a,b,d^	Look and feel includes colors from activity guidelines and those familiar within the UK National Health Service.
	Personalization of app ^a,b,d^	User guide will be developed. Users can increase/decrease the font size of the educational sections as required. Content delivered included “personas” relevant to a Scottish context.
	Integration of reminders ^a,b,d^	Users can set reminders any time through clicking on planned activity. A prompt will be given to users when originally setting an event.

^a^Based on co-design discussions.

^b^Based on UK activity guidelines.

^c^Based on models of behavioral change.

^d^Based on user design principles.

#### Physical Activity Elements

Our proposed methods for recording muscle and balance activity by number of days completed were well received by participants: 67% found the approach “very suitable” and 33% found the approach “somewhat suitable.” We had similar responses for our approach to measure cardiovascular activities using “time” and “intensity,” where 60% of respondents found the approach to be “very suitable.” For sedentary breaker activities, all respondents wanted these to be accessible from both the educational and physical activity sections of the app. In terms of the “look and feel” of the instructional videos, there was no consensus on whether to use university branded clothing or casual (without branding) clothing while giving instructions. Unfortunately, the link to a prototype video had stopped working for 3 users by the time several of the questionnaire respondents completed their feedback, and therefore we could not explore the responses for this question.

#### Educational Elements

During co-design online meeting 3, we presented full draft sections of the educational elements, overall structure, and proposed rules for development (eg, developing some standards/formatting requirements). In our follow-up questionnaire, we asked participants how relevant the educational elements were for our target group, where 83% of respondents stated that the content was “very relevant.” In terms of usability, 67% of the respondents deemed that the educational materials as presented were “very user friendly,” and 33% thought they were “a little user friendly.” Three participants gave further feedback that the app should incorporate carer *“*experience and voices,*”* and participants could see value in the developments that had taken place since the previous development sprints: “You can see it developing and coming together from previous stages. This is much improved.” Participants encouraged the use of audio delivery of materials alongside visual presentation of materials such as images and videos. Other feedback included that the number of (optional) interactive tasks was “the right amount” for 50% of respondents (Figure 6D).

#### Communication Elements

After analyzing feedback from participants, we decided to give priority to the implementation of the educational and physical activity elements over communication elements. Participants were asked what sort of information they would like to share on social media in future versions of the app. A variety of communication mechanisms were suggested, such as forums (eg, “In future, I think a forum where they can support each other or a buddy system would be beneficial”); use of emojis (“How you feel after exercise such as smiley faces/thumbs up, small bite size text such as a Twitter-style link, or share with friends family or other social media, challenge others”)*;* and finally progress-sharing mechanisms (eg, “I think the progress page is suitable to be able to share”).

### Implementation of Design Sprint 3

Our last development sprint finalized the app design as requested by the group. We made every effort to address any aspects for which clear and feasible changes had been requested by our co-design group. A major focus of this sprint was the creation of the physical activity videos, taking place from the home setting by an “Active Lifestyle” officer based at the university. Videos developed were no more than 5 minutes long, considering both the stage of change and lack of free time of caregivers. These videos (accessible via the planner) were integrated into the final app version. All team members were involved in testing the final app functionality. Using a task checklist, we evaluated elements of consistency, error prevention, and clarity. Although many of these passed user testing, we did notice that the code added to allow font resizing as an accessibility feature failed on some phones, and the videos displayed were too small on others. This reinforced the need for extensive testing on a wide range of devices and, in the event that something is missed, we put in place ongoing procedures to update the software.

### Final Prototype Developed

The CareFit final prototype (see Figure 7 for screenshots) was designed to be used for the duration of a 3-week study. Users could navigate to the different parts of CareFit via the following main menu options: Weekly Planner, Education, Reminders, Share Progress.

The Weekly Planner allowed planning of physical activities for up to 2 weeks ahead. Users could also view activities planned and completed during the previous week. The planner allowed users to plan up to three types of physical activities (with a bespoke icon and individual screen for each) on any day of the week. When users were unable to complete an activity as intended, they had the ability to move the activity to another date of their choice. CareFit users could choose from the following types of physical activities based on current guidelines: (1) cardiovascular activities plus a daily activities option where the activity took place outside of the app-delivered elements (where the user could set the intensity and duration level and/or use custom activities); (2) muscle and balance activities (where the user sets the intensity level); and (3) three sedentary breaker activities that users were free to choose from.

Instructions on how to perform exercises were delivered via videos hosted on YouTube. The videos were focused on developing functional fitness while acknowledging daily life constraints imposed by being a caregiver. The education section was structured as follows: (1) Welcome and Introduction, (2) Physical Activity: Beginners Guide, (3) Relationships and Physical Activity, (4) Managing Time, (5) Goals and Rewards, (6) Physical Activity and Consequences, (7) The Mind and Body, and (8) Knowledge Quiz. The reminders section of the app let users manage reminders for activities they had planned. Once a reminder was set in the planner, users could use the reminders section to view their reminders or delete unwanted reminders. The “Share progress” functionality let users share a summary image of physical activities/achievements completed to be shared across a variety of social media/phone platforms.

## Discussion

### Principal Findings

Regular physical activity is important for everyone; however, many groups are underserved by existing guidance and targets [26]. Globally, we lack sustainable formats for the delivery of physical activity instructions for those on the lower end of the physical activity level spectrum [27]. Caring responsibilities can push individuals needlessly toward becoming a “syndemic” statistic (ie, being vulnerable due to the effects of widespread noncommunicable disease) [6], including cases where individuals lack the time, tools, or motivation to undertake regular physical activity. Cumulative data from more than 80,000 people and 64 studies suggest that the COVID-19 pandemic has been associated with an increase in sedentary behavior and a decrease in physical activity [28], where lack of physical activity (and its associated effects) will remain a critical concern for chronic disease [29-31]. It is not simply the risk of mortality or poor health from future pandemics that is of concern, but it is also the seemingly inevitable poor quality of life, deterioration of health, hospitalizations, and other crisis points that can affect both the carer and those cared for [6,7]. Perhaps one of the most striking lessons of the COVID-19 pandemic is that caregivers are irreplaceable. Here, we have presented a rapid response project that is a first in digital health: a prototype app co-designed by carers that delivers a personalized approach to behavioral change science aimed at improving physical activity in the home.

The development of this app offers several opportunities for further learning. The use of co-design in caregiver research is growing and aligns well with other emerging work. Our strategy was to equip our participants with a variety of different stakeholder viewpoints through discussion before completing questionnaires [32]. Such co-design was successfully used previously by Xu et al [32] when designing an app for caregivers of children with atopic dermatitis to develop functionalities such as login, disease diary, journal, chatbot, forum, and disease monitor. As part of this work, participants helped us to identify several different barriers and enablers to physical activity from the home, including lack of time, finding a way to recognize efforts, and being able to conduct activities safely. Similar findings have been replicated elsewhere both in physically active and inactive populations [33,34]. For example, Hoare et al [33] surveyed a total of 894 Australian adults aged 25 to 54 years, who were both active and inactive, and found that lack of time, lack of enjoyment, and a preference to do other things were key barriers toward physical activity. Mulligan et al [34] systematically searched for personal barriers of physical activity participation for people with neurological conditions, and found that safety, confidence, and lack of support were key contributors to lack of physical activity.

Our results demonstrate the utility of online co-design: carers and care professionals have made measurable contributions to the project at every stage of the design process, taking the app from a “fuzzy” concept to the implementation and evaluation stages [20]. A key theme (and enabler) within the app design is to value the role of the carer within the framework of activity guidelines (eg, a few minutes of activity is better than none), and to recognize that common caring activities such as cleaning, lifting, and moving have inherent value for physical health [18,35]. We have designed the app wherever possible to be supportive. There is no pressure put on the carer to undertake physical activity, and personalization is possible through making individual plans, exercises, and engaging with the education sections as and when required. We also supported caregiving tasks wherever possible (eg, lifting, carrying). Simplicity of the design (both in terms of content and technology use/delivery) is a core element of the solution. Physical activity guidelines and behavioral change models are distilled into manageable, daily tasks.

The theoretical underpinnings of this app are of considerable interest to future work and practice. Our use of the TTM allowed for several personal reflective exercises to be developed that were suited to the stage of change our participants were at (eg, goal setting and list of pros and cons). We are not the first to develop elements of the TTM into a digital app. There is evidence to suggest that this model of behavioral change can allow up to 6 months of positive behaviors within a “GreyMatters” app study [36]. The context of the study was to support individuals with healthy lifestyle factors that reduce the chances of developing dementia (eg, targeting holistic health needs across cognition, diet, physical health, sleep, social, and stress). App use was supported by a coach that incorporates both personal and simplified generic goals. Although there are similarities with CareFit (including scope to expand CareFit to support more holistic health care needs), the populations served by these apps remain largely distinct. While the design of the app aligns well with the TTM overall, the precise modality of interaction that works best now needs to be researched further. For example, previous literature has shown that goal setting is not straightforward, and certain app features such as “trophies” and “ribbons” in themselves are insufficient to motivate participants to undertake physical activity on a regular basis [37]. Further complicating matters is that components of the UK national physical activity guidelines can be difficult to put into action. There is no specific “dose” of muscle and balance activity work, only a recommendation that the activity should take place 2 days a week. Future related work could explore other stages of the TTM (eg, action stage) in greater depth, including over a longer duration (more than 3 weeks). There are also future options to expand CareFit by integrating wearable technology, supporting further outdoor activities, and increasing educational information available. Other interesting areas for future exploration include understanding how individuals can be supported in undertaking exercises correctly and how the app could identify those who are most at risk of complications from being overweight/obese [38]. Finally, the digital divide remains a significant risk to reaching the caregiver population, which must be accounted for [39].

There are some limitations of note relating to this work. CareFit was developed as a rapid response to COVID-19 (6-month project duration) in the middle of a global pandemic where convenience sampling may have skewed our feedback. Participant engagement was structured to genuinely collate the opinions of our co-design participants; however, prioritization through online MoSCoW methodology with supporting online meetings is not infallible [40]. Further research is required to test the external validity of our approach. Despite the short timeframe of this project, we managed to integrate many requirements stemming from participants’ feedback. However, combining different sources of information still requires researcher-based decision-making. The evidence-based materials used (eg, behavioral change, government guidelines, educational activities) have not been synthesized and delivered in this manner before, and the extent to which individual caregivers can guide themselves through the materials needs further appraisal. Not least is the barrier of caregivers being left with “another” task in their busy schedules: physical activities may work best where unmet needs are addressed holistically [41]. Our users did not extensively test the final prototype built, as our focus for such questions is reserved for a real-world trial.

### Key Lessons and Future Recommendations

Key lessons from this work are as follows. Primarily, this work emphasizes the value of the co-design process and the importance of involving carers and care professionals in research and practice. In addition, the feasibility of co-designing evidence-based physical activity apps for caregivers with a small development team is demonstrated, even with the limitations imposed by COVID-19 restrictions. Our results also highlight the importance of synergy among theory, expert knowledge, and target users’ personal experience in developing bespoke solutions for special populations such as caregivers. The need for assistive technologies to move from computer solutions to portable device–based solutions is further emphasized. We have also shown that developing a user-centered digital health app to improve the quality of life of caregivers is feasible. Nevertheless, the digital literacy of caregivers will vary significantly, and further exploration will be needed to understand what works in practice in terms of confidence and support. There are also gaps in current knowledge regarding physical activity guidelines to be addressed, such as whether caregivers are receiving information and how to measure components objectively. The constraints of the Android environment can be a limitation to user experience, especially with respect to difficulties in updating app versions. Overall, feedback from our participants demonstrates the strength of the co-design process as opposed to universal design apps.

### Conclusion

We have demonstrated the utility of the co-design process to develop a novel approach to combine national physical guidelines and behavioral change models into a personalized app for carers. Further work is now required to explore the acceptability, usability, and feasibility of this app within a real-world setting.

## Appendix

Multimedia Appendix 1 Example of the first co-design postdiscussion survey.

## Abbreviations

FURPS+: Functional, Usability, Reliability, Performance and additional requirements
MoSCoW: “Must Have, Should Have, Could Have, Won't Have this time” prioritization technique
TTM: transtheoretical model of behavioral change
