# Supplementary material for: A Novel Mobile App (“CareFit”) to Support Informal Caregivers to Undertake Regular Physical Activity From Home During and Beyond COVID-19 Restrictions: Co-design and Prototype Development Study
Source: JMIR Form Res. 2021 Oct 1;5(10):e27358. doi: 10.2196/27358 (PMC8489565; doi:10.2196/27358)
Supplement: Multimedia Appendix 1 [file formative_v5i10e27358_app1.docx]

# Appendix 1: Example of First co-design post discussion survey

Carefit co-design session 1: individual feedback form

**For filling out after your first co-design session/interview:**
 
*The purpose of this form is to understand more detail in the views of the group following our discussions. It is designed to take no longer than 15 to 20 minutes to complete.*
 
*For reference, a simple video demonstration of the prototype* application*can be found here:*

Section 1: Understanding our core app functions

1.1 Briefly, what helps or prevents unpaid carers staying physically well?

1.2 Briefly, where do you see the role for technology to support physical health for carers?

1.3 Briefly, describe the useful/core functions of other applications that you use that you would like to see in Carefit:

## Section 2: Critique of the existing prototype

What do you think we should keep/lose/change about the initial prototype?

1.4 Keep:

1.5 Lose

1.6 Change:

Section 3: App delivery and prioritisation exercise
 
Building on our first codesign discussion, this part of the survey is to help us prioritise our focus within the application development. Please select whether you think an element is something that we:
 
- Won't have (i.e. in this version of the app)
- Could have;
- Should have; or 
- Must have.
 
The exercise is meant to be built around your instincts so the intention is that you wouldn't spend a lot of time on any individual question. If you have additional ideas for the application, these can be shared at the end of this section.

1.7 Educational elements (to read/static components)

|  | Wont have (1) | Could have (2) | Should have (3) | Must have (4) |
| --- | --- | --- | --- | --- |
| Quotes of the day/tips (1) |  |  |  |  |
| Short modules (e.g. based around the trans-theoretical model and behavioural change alongside government guidelines) (2) |  |  |  |  |
| Reminders of educational elements (e.g. after completion) (3) |  |  |  |  |

1.8 Educational elements (to do/functional components)

|  | Wont have (1) | Could have (2) | Should have (3) | Must have (4) |
| --- | --- | --- | --- | --- |
| Questionnaire on current physical activities (e.g. to understand your starting point aligned government guidelines and the trans-theoretical model of behavioural change) (1) |  |  |  |  |
| Pros and cons list for undertaking physical activities (aligned to the trans-theoretical model of behavioural change) (2) |  |  |  |  |
| Time planning exercise (e.g. captures existing activities (including "active living" and plans future activities across the week as a reflection exercise) (3) |  |  |  |  |
| Weekly planner (e.g. builds on other components to plan out week ahead and promote behavioural change) (5) |  |  |  |  |
| Triggers and relapse exercise (e.g. planning for relapse in your plan- this information could be relayed to participants at the point of relapse) (6) |  |  |  |  |
| Meditation exercise (Step by step instructions for a simple meditation such as breathing) (7) |  |  |  |  |
| Quiz (used to reinforce learning of all educational elements) (8) |  |  |  |  |

1.9 Motivational elements

|  | Wont have (1) | Could have (2) | Should have (3) | Must have (4) |
| --- | --- | --- | --- | --- |
| Targets (short term/day to day) (1) |  |  |  |  |
| Daily questions (e.g. tracking progress) (2) |  |  |  |  |
| Routine builders/reminders (e.g. prompting engagement with the process) (3) |  |  |  |  |
| Identifying improvements/positivity (e.g. developing the app in a way that allows positive, personalised feedback) (4) |  |  |  |  |
| Goal setting (e.g. for each week) (5) |  |  |  |  |
| Physical test 1: Step test (e.g. Step on and off the box for three minutes) (6) |  |  |  |  |
| Physical test 2: 6 minute walk test (e.g. measures the distance an individual is able to walk over a total of six minutes on a hard, flat surface) (7) |  |  |  |  |
| Physical test 3: Sit to stand test (e.g. sitting and standing over 30 seconds to test leg strength and endurance) (9) |  |  |  |  |

1.10 Reviewing feedback

|  | Wont have (1) | Could have (2) | Should have (3) | Must have (4) |
| --- | --- | --- | --- | --- |
| Times (total time spend undertaking physical activity) (1) |  |  |  |  |
| Sedentary time (total time spent sedentary) (12) |  |  |  |  |
| Reps (e.g. number of times you perform a specific exercise) (2) |  |  |  |  |
| Sets (e.g. number of cycles of reps that you complete) (10) |  |  |  |  |
| Daily streak (e.g achievements over a number of days) (3) |  |  |  |  |
| Government guidelines (e.g. prompts and feedback) (4) |  |  |  |  |
| Rate of perceived exertion (e.g. how tough did you find the exercise) (5) |  |  |  |  |
| How you feel after each exercise (e.g. your mood) (11) |  |  |  |  |
| Frequency, intensity, type and time (e.g. your overall physical activity programme across days/weeks in terms of how often, how hard you want to work, what your doing and how long for) (6) |  |  |  |  |

1.11 Physical activities

|  | Wont have (1) | Could have (2) | Should have (3) | Must have (4) |
| --- | --- | --- | --- | --- |
| Cardiovascular focused (e.g. walking, cycling) (1) |  |  |  |  |
| Sedentary focused (e.g. reducing extended periods of sitting/breaking up watching tv with movement etc) (2) |  |  |  |  |
| Muscle Strength (e.g weight lifting- tin of beans in the kitchen, carrying a shopping bag) (3) |  |  |  |  |
| Muscle Endurance (e.g. using muscles for a prolonged period of time such as gardening, housework) (4) |  |  |  |  |
| Flexibility (e.g. Stretching, Yoga) (5) |  |  |  |  |
| Body composition (weight management- body fat) (6) |  |  |  |  |

1.12 Personalisation features

|  | Wont have (1) | Could have (2) | Should have (3) | Must have (4) |
| --- | --- | --- | --- | --- |
| Name of carer (1) |  |  |  |  |
| Name of person cared for (2) |  |  |  |  |

1.13 Communication

|  | Wont have (1) | Could have (2) | Should have (3) | Must have (4) |
| --- | --- | --- | --- | --- |
| Coach (e.g. a sports professional) (1) |  |  |  |  |
| A message board (all other users of the app) (6) |  |  |  |  |
| Buddy (e.g. another carer using the application) (2) |  |  |  |  |
| Family member/Friend (e.g. sharing progress on social media) (3) |  |  |  |  |
| Challenge others (e.g. other caregivers) (4) |  |  |  |  |

1.14 Type of presentations required for education and activity elements

|  | Wont have (1) | Could have (2) | Should have (3) | Must have (4) |
| --- | --- | --- | --- | --- |
| Video (1) |  |  |  |  |
| Visual (2) |  |  |  |  |
| Written instructions (3) |  |  |  |  |
| Audio (4) |  |  |  |  |

1.15 Please share any further comments you may have on the core components of the app here:

## Section 4: App delivery and priorization exercise

1.16 How best should we capture/measure physical activity progress in our participants (within a timeframe of 2 to 3 weeks)?

1.17 What are appropriate goals for caregivers across the three domains of: (i) cardiovascular exercise, (ii) sedentary behaviour, (iii) flexibility /strength training?

1.18 How many different activities would you like to see in the application (i.e how many individual activities to choose from such as "sit to stand")?

1.19 What do you think is the "right level" of support for participants to stay physically safe using the app without affecting motivation?

|  | First log in- digital (1) | Every log in- digital (2) | Multiple reminders- digital (3) | Paper based (e.g. study consent form/information sheet) (4) | Not required (5) |
| --- | --- | --- | --- | --- | --- |
| Disclaimer about risk of injury (1) |  |  |  |  |  |
| Physical Activity Readiness Questionnaire (or equivalent measures that may result in the suggestion of a referral to a GP) (2) |  |  |  |  |  |
| Brief summary information about safety (8) |  |  |  |  |  |
| Bespoke advice for how to do each exercise safely (4) |  |  |  |  |  |
| Other (10) |  |  |  |  |  |

1.20 If you have any other comments about physical safety- please provide them here

1.21 Finally, adding up to a total of 100% between all three components- what type of balance of focus would you ideally like to see in the application across education, physical activity and community?
 
NB- Please make the combined percentage of [education] + [community]+ [physical activity] = 100%

|  | Percentage |
| --- | --- |

|  | 0 | 10 | 20 | 30 | 40 | 50 | 60 | 70 | 80 | 90 | 100 |
| --- | --- | --- | --- | --- | --- | --- | --- | --- | --- | --- | --- |

| Education (e.g. themes around motivation, government guidelines and sustaining behavioural change) () | 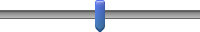 |
| --- | --- |
| Community (e.g. connecting to others such as a physical activity coach, family members, friends etc) () | 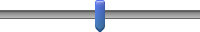 |
| Physical activity exercises (e.g. variety and depth) () | 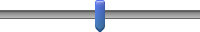 |

1.22 If you have any other comments you would like to
